# Tranexamic acid efficacy in geriatric hip fractures: impact of nutritional status on blood loss, transfusion rates, and safety

**DOI:** 10.1186/s12891-024-07665-x

**Published:** 2024-07-17

**Authors:** Jun Xie, Shinkichi Himeno

**Affiliations:** Department of Orthopedic Surgery, Himeno Hospital, 2316 Nishiro, Hirokawa Machi, Fukuoka Prefecture, 834-0115 Japan

**Keywords:** Tranexamic acid, Hip fracture, Nutrition, Transfusion rate, Blood loss

## Abstract

**Background:**

Tranexamic acid (TXA) is a widely employed intervention in orthopedic surgeries to minimize blood loss and the need for postoperative transfusions. This study focuses on assessing the efficacy and safety of TXA specifically in undernourished older adults undergoing hip fracture procedures.

**Methods:**

A total of 216 patients were classified into two groups based on the Geriatric Nutritional Risk Index: undernourished and normal. In total, 82 patients received intravenous TXA at a dosage of 15 mg/kg before incision, with an additional 1 g administered intravenously over a 3-hour period postoperatively. Postoperative hemoglobin (Hb) drop, blood transfusion rate, and the incidence of deep venous thrombosis (DVT) were assessed in each group according to the presence or absence of TXA. Additionally, demographic factors including age, sex, body mass index, and serum albumin were investigated.

**Results:**

51.9% patients were identified as undernourished, experiencing progressive anemia (Hb: 10.9 ± 1.5 g/dL) and hypoalbuminemia (serum albumin: 31.9 ± 8 g/L). In comparison with the normal group, undernourished individuals were more likely to sustain femoral neck fractures (undernutrition vs. normal: 56.2 vs. 42.3%) and less likely to incur trochanteric fractures (undernutrition vs. normal: 43.8 vs. 57.7%) (*P* = 0.043). TXA administration significantly reduced the transfusion rate (*P* = 0.014) and Hb drop (*P* = 0.001) in the normal nutritional group, while its impact on the undernourished group remained less pronounced. There was no significant association between TXA administration and the rate of DVT complications, irrespective of the nutritional status.

**Conclusions:**

Undernutrition not only diminishes muscle strength and gait function, leading to various types of hip fractures, but it may also hinder the efficacy of TXA in reducing blood transfusion rates and blood loss.

## Background

Hip fractures stand as a prominent cause of morbidity and mortality among older adults, often necessitating surgical intervention. Perioperative blood loss emerges as a common complication, frequently leading to the need for transfusions. This poses risks such as immunological reactions, disease transmission, heightened susceptibility to surgical site infections, and prolonged hospital stays [1, 2]. In the pursuit of mitigating these complications, various blood-sparing techniques have been explored. Notably, tranexamic acid (TXA) has garnered attention for its reported effectiveness and safety in reducing both blood loss and postoperative transfusion rates across a diverse range of clinical scenarios [3–5].

Furthermore, older individuals exhibit a heightened prevalence of undernutrition, with up to 60% of those experiencing hip fractures falling into the undernourished category. This undernutrition contributes to a spectrum of postoperative complications, including prolonged hospital stays, increased likelihood of readmission, compromised mobility, and the failure of internal fixation [6–8]. Moreover, undernutrition serves as a risk factor associated with nutrient deficiency-related anemia [9]. This connection leads to a gradual decline in hemoglobin (Hb) levels due to additional bleeding from the fracture site, necessitating blood transfusions during the perioperative period.

Nevertheless, the effectiveness and safety of TXA administration in undernourished patients remain uncertain. In light of this uncertainty, the present study was formulated to explore the correlation between nutritional status and the efficacy of TXA in mitigating perioperative blood loss and reducing transfusion rates among older adults undergoing hip fracture procedures.

## Methods

### Ethics approval

This study adhered to the ethical principles outlined in the Declaration of Helsinki. It was retrospectively registered and received approval from the ethics committee review board of Himeno Hospital (No. 22R-02). Informed consent was obtained from all participants.

The study included 216 geriatric patients (≥ 65 years) admitted with hip fractures, encompassing femoral neck and trochanteric fractures resulting from accidental falls, during the period from April 2019 to June 2022. Before the day of surgery, comprehensive data, including Hb, serum albumin, body mass index (BMI) were recorded. Patients on systemic anticoagulation, including the use of warfarin, direct oral anticoagulants such as dabigatran, rivaroxaban, apixaban, and edoxaban, as well as antiplatelet medications like clopidogrel and aspirin, were identified and promptly discontinued upon admission.

Exclusion criteria comprised patients with severe preoperative anemia necessitating transfusion (Hb < 7 g/dL), those diagnosed with deep venous thrombosis (DVT) through lower limb Doppler ultrasound examination before the surgery, and individuals with significant kidney dysfunction indicated by a glomerular filtration rate < 60 ml/min/1.73 m^2^. Although epilepsy was a contraindication for TXA use, no patients with epilepsy were hospitalized during the study period due to the small size of our hospital. The nutritional status of patients was assessed using the Geriatric Nutritional Risk Index (GNRI), calculated as GNRI = 1.489 × serum albumin (g/L) + 41.7 × (weight/ideal weight). Ideal weight was determined using the Lorenz equation for men (height (cm) – 100 - [(height (cm) – 150)/4]) and women (height (cm) – 100 - [(height (cm) – 150)/2.5]) [10]. Patients with GNRI values exceeding the cutoff of 92 were categorized as the normal group, while those with values ≤ 92 were classified into the undernutrition group [10, 11].

All surgeries were conducted within 24–48 h after admission, following the enhanced recovery after surgery protocol implemented in our hospital. For surgical techniques, trochanteric fractures underwent intramedullary nail fixation, while femoral neck fractures were treated with hemiarthroplasty. No patients underwent total hip arthroplasty during the study period. Although total hip arthroplasty can offer excellent outcomes for certain patients, factors such as functional requirements, life expectancy, mobility goals, surgical risk, bone quality, and complication rates were considered. This led to the preference for less invasive and less demanding procedures in our patient population. No drains were utilized in any surgical procedure. Surgical time and blood loss during the surgery were also recorded.

In this retrospective study, the administration of TXA commenced for 82 patients starting from October 2020. From April 2019 to September 2020, a control group comprised of 134 patients who did not receive TXA was established, irrespective of their nutritional status or fracture type. Consistent with prior protocols, the initial TXA dose (15 mg/kg) was intravenously administered 15 min prior to incision, followed by a continuous postoperative infusion of a second dose (1 g) for the subsequent 3 h [12]. Edoxaban tosilate hydrate (15 mg/day), a direct-acting oral anticoagulant, was routinely administered on postoperative day 1 and continued for 14 days.

Hemoglobin levels were assessed both preoperatively and on the first postoperative day only, due to the relatively short half-life of TXA. The percentage decrease in Hb calculated as (preoperative Hb – postoperative Hb)/preoperative Hb×100%, was considered as Hb drop. Blood examinations were conducted weekly for up to 2 weeks postoperatively. Transfusion criteria typically indicated Hb levels below 7 g/dL. For patients with cardiovascular risk factors, transfusion was recommended at Hb levels below 8 g/dL, in accordance with clinical guidelines. However, due to limitations in our hospital’s capacity and the unavailability of a cardiovascular physician, some patients with significant cardiovascular risk factors were not hospitalized. In cases where Hb levels ranged from 7 g/dL to less than 10 g/dL, the decision to transfuse was based on the presence of persistent symptoms such as lightheadedness, orthostatic hypotension, or tachycardia during the perioperative period. To monitor potential complications associated with TXA, all patients underwent ultrasonography screening for DVT on the seventh postoperative day.

### Statistical analysis

For comparative analysis of age, pre- and postoperative Hb levels, Hb drop, BMI, serum albumin, surgical time, and intraoperative blood loss between groups, Student’s *t*-test was applied. *Fisher’s* exact test was utilized to assess sex, fracture type, blood transfusion rate, and the incidence of DVT. All statistical analyses were carried out using EZR software version 4.0.3 (Copyright (C) 2020; The R Foundation for Statistical Computing) [13]. Statistical significance was defined at *P* < 0.05.

## Results

### Patient demographics

A total of 216 older adults participated in this study, comprising 187 women and 29 men. Among them, 109 patients underwent intramedullary nail fixation for trochanteric fractures, while 107 underwent hemiarthroplasty for femoral neck fractures. Based on the Geriatric Nutritional Risk Index (GNRI), 112 patients (51.9%) were categorized into the undernutrition group, and 104 patients (48.1%) into the normal nutrition group. Table 1 presents the patient characteristics and corresponding statistical values.

**Table 1:** Patients’ characteristics

Variable	Undernutrition (*n* = 112)	Normal (*n* = 104)	*P*-value
Age (Years)	86 ± 7.9	87 ± 7.2	0.383
Gender	Women 94 (84%) Men 18 (16%)	Women 93 (89%) Men 11 (11%)	0.318
BMI (kg/m^2^)	18 ± 2.8	21.8 ± 3.2	< 0.001
TXA usage (%)	33	43.2	0.126
Serum albumin (g/L)	31.9 ± 8	39.6 ± 3.1	< 0.001
Preoperative Hb(g/dL)	10.9 ± 1.5	11.7 ± 1.3	< 0.001
Fracture type (%)	TF 43.8 FN 56.2	TF 57.7 FN 42.3	0.043

TXA: tranexamic acid

Hb: hemoglobin

TF: trochanteric fracture

FN: femoral neck fracture

No significant differences were observed between the two groups regarding age, sex, and the rate of TXA administration. However, preoperative Hb level, serum albumin level, and BMI were significantly lower in the undernutrition group compared to the normal group (undernutrition vs. normal: Hb, 10.9 ± 1.5 vs. 11.7 ± 1.3 g/dL, *P* < 0.001; serum albumin, 31.9 ± 8 vs. 39.6 ± 3.1 g/L, *P* < 0.001; and BMI, 18 ± 2.8 vs. 21.8 ± 3.2 kg/m^2^, *P* < 0.001). Additionally, there was a higher incidence of trochanteric fractures and a lower incidence of femoral neck fractures in the normal nutrition group compared to the undernutrition group (normal vs. undernutrition: trochanteric fracture, 57.7% vs. 43.8%; femoral neck fracture, 42.3% vs. 56.2%; *P* = 0.043).

### Primary outcomes

The primary outcomes following TXA administration in the two groups are elaborated in Table 2. Within the normal group, TXA administration was strongly associated with a reduction in Hb drop resulting from a higher postoperative Hb level (*P* = 0.001), and less transfusion rate (*P* = 0.014). In contrast, TXA administration in the undernutrition group showed no statistical differences in Hb drop and transfusion rate, as well as other factors including age, intraoperative blood loss, pre or postoperative Hb levels, and surgery time. Comparison of the effectiveness of TXA between undernourished and typical patients was also compared. Among the patients receiving TXA administration, there were 37 undernourished and 45 normal patients. A significantly reduced transfusion rate (8.9%) was noted in normal patients compared to undernourished patients (32.4%, *P* = 0.011).

**Table 2:** Primary outcomes after administration of TXA in undernutrition and normal nutrition groups

Variable	Undernutrition		*P*-value	Normal		*P*-value
	TXA(-) (n = 71)	TXA(+) (n = 35)		TXA(-) (n = 54)	TXA(+) (n = 41)	
Surgery Time (min)	59.7 ± 22	51.3 ± 27.7	0.092	55.3 ± 18.4	47 ± 26.9	0.101
Intraoperative Blood Loss (mL)	186.6 ± 163	143.3 ± 122.1	0.248	170.3 ± 140.5	151.8 ± 118.3	0.477
Transfusion Rate (%)	28	32.4	0.663	28.8	8.9	0.014
Preoperative Hb(g/dL)	10.8 ± 1.5	11.1 ± 1.6	0.432	11.8 ± 1.5	11.8 ± 1.0	0.875
Postoperative Hb(g/dL)	8.4 ± 1.2	8.9 ± 1.9	0.087	8.9 ± 1.9	9.7 ± 1.5	0.024
Percentage Hb Drop (%)	22.2 ± 9.3	19.9 ± 11.3	0.248	25.4 ± 10.2	18.7 ± 9.2	0.001
Fracture Type (%)	TF 48 FN 52	35 65	0.228	TF 62.7 FN 37.3	51.1 48.9	0.317
DVT rate (%)	1.4	0	1.0	3.7	2.4	1.0

TXA: tranexamic acid

Hb: hemoglobin

DVT: deep venous thrombosis

### Incidence of DVT

Among the participants, one patient in the undernutrition group and two patients in the normal group (without TXA administration), along with one patient in the normal group (with TXA administration), experienced DVT. However, no instances of pulmonary embolism were reported in either group. Notably, there was no significant association between TXA administration and the rate of DVT complications, irrespective of the nutritional status.

## Discussion

TXA has gained widespread acceptance in geriatric hip fracture cases for its efficacy and safety in reducing bleeding and postoperative transfusions, attributed to its potent antifibrinolytic properties [14, 15]. Concurrently, geriatric nutritional challenges, such as undernutrition, sarcopenia, and frailty, exert substantial impacts on disability, complication rates, and mortality following hip fractures [9]. Despite these well-established aspects, the impact of TXA on blood loss and transfusion rates in the context of undernutrition, prevalent in older adults, remains unclear. To our knowledge, this study represents the inaugural examination focusing on the effect and safety of TXA in patients with hip fractures under varying nutritional statuses.

Undernutrition is characterized by an imbalance between the body’s energy intake and requirements. A prior study indicated the prevalence of undernutrition in 30–70% of hospitalized older adults with hip fractures [16], aligning well with our findings (51.2%). In our study, undernourished patients exhibited a higher incidence of femoral neck fractures and a lower incidence of trochanteric fractures compared to normally nourished patients. This suggests that impaired muscle function resulting from undernutrition may contribute as a risk factor for the type of fracture sustained. Supporting this notion, Yerli et al. investigated the impact of hip muscle attenuation on hip fracture type and concluded that both muscle mass and weakness may influence fracture type [17]. Additionally, another study reported a notable association between total gluteus maximus volume and femoral neck fractures [18]. Consequently, further interventional studies are warranted to explore the specific considerations and interventions required for undernourished patients with hip fractures.

In comparison with the normal group, the impact of TXA on undernourished patients was limited, exhibiting less effect in reducing the transfusion rate and Hb drop. Undernourished status may have altered pharmacokinetics, including changes in drug absorption, distribution, metabolism, and excretion. These alterations could affect the bioavailability and efficacy of TXA, leading to reduced effectiveness in controlling blood loss [19, 20]. Additionally, undernutrition can be associated with alterations in the coagulation cascade, including changes in fibrinolytic activity. It’s possible that undernourished patients have a less pronounced fibrinolytic response to surgery, reducing the potential benefit of TXA in inhibiting fibrinolysis [21]. Therefore, the association between the effects of TXA with preoperative coagulation parameters should be investigated in further studies. Moreover, undernourished patients may have compromised hemostatic reserves due to nutritional deficiencies, particularly in essential nutrients like vitamin K, iron, and folate. These deficiencies could impair the body’s ability to form stable blood clots, limiting the efficacy of TXA in preventing excessive bleeding [22]. Finally, undernutrition can impair wound healing processes, including collagen synthesis and tissue repair [23]. TXA primarily works by stabilizing fibrin clots to reduce bleeding, but if wound healing is compromised in undernourished patients, the efficacy of TXA may be diminished. Thus, a comprehensive approach to malnutrition treatment, beginning with accurate diagnosis and identification of underlying conditions, is imperative before surgery to minimize transfusion rates.

Regarding TXA, the foremost concern is the potential risk of DVT. However, our study observed a low DVT rate, and importantly, no association with TXA administration was identified. This implies that TXA is a safe approach for older adults with varying nutritional statuses.

This study has several limitations. Firstly, its retrospective design may introduce inherent biases. Additionally, the limited sample size increases the risk of statistical error, compounded by the absence of a formal power analysis, which may affect the robustness of our findings, particularly for smaller subpopulations. Further studies, including a multicenter approach, are necessary to achieve a larger sample size within a shorter investigation period, especially considering the medical care capacity of a single hospital, such as ours with fewer than 150 beds. More comprehensive control for confounding variables, such as nutritional status, comorbidities, coagulation parameters, and surgical techniques, should also be prioritized to provide a more nuanced understanding of TXA’s effects in this population. Notably, the lack of systematic recording of preoperative coagulation parameters is a significant limitation, as variations in these parameters can significantly influence surgical outcomes and TXA efficacy. Future studies should include a comprehensive preoperative coagulation work-up to better understand the influence of these parameters on the outcomes.

Given the variability in bleeding risk depending on the severity and subtype of hip fractures, incorporating comparisons based on detailed fracture subtypes into the study design would provide valuable insights. It is worth noting that the evaluation of postoperative Hb drop was confined to the first postoperative day. TXA has a short half-life of 2 to 3 h, with hemostatic effects lasting 6 to 8 h. While this study focused on the immediate impact of TXA, including routine lab controls throughout the postoperative period would provide a more comprehensive understanding of the hemostatic status and the prolonged effects of TXA. This is particularly important for malnourished patients who may exhibit prolonged bleeding due to compromised hemostatic reserves. Future studies should incorporate these additional data points to enhance the robustness and clinical relevance of the findings.

## Conclusions

Undernutrition is highly prevalent among older adults with hip fractures, influencing not only muscle strength and gait function but also contributing to different types of hip fractures. Moreover, undernutrition may impede the efficacy of TXA in reducing blood loss and transfusion rate. Therefore, early nutritional intervention is imperative to mitigate the occurrence of hip fractures, associated complications, and mortality, while also shortening the duration of rehabilitation.

## Data Availability

The datasets used and analyzed in the current study are available from the corresponding author on reasonable request.
